# Supplementary material for: Subsurface ocean warming preceded Heinrich Events
Source: Nat Commun. 2022 Jul 21;13:4217. doi: 10.1038/s41467-022-31754-x (PMC9304376; doi:10.1038/s41467-022-31754-x)
Supplement: Supplementary file 1 — Supplementary Information [file 41467_2022_31754_MOESM1_ESM.pdf]

Supplementary Materials for:

Subsurface ocean warming preceded Heinrich Events

L. Max*, D. Nürnberg, C. M. Chiessi, M. M. Lenz, S. Mulitza

This supplementary document contains the following information:

Supplementary Figs. 1 to 4

Supplementary Table 1

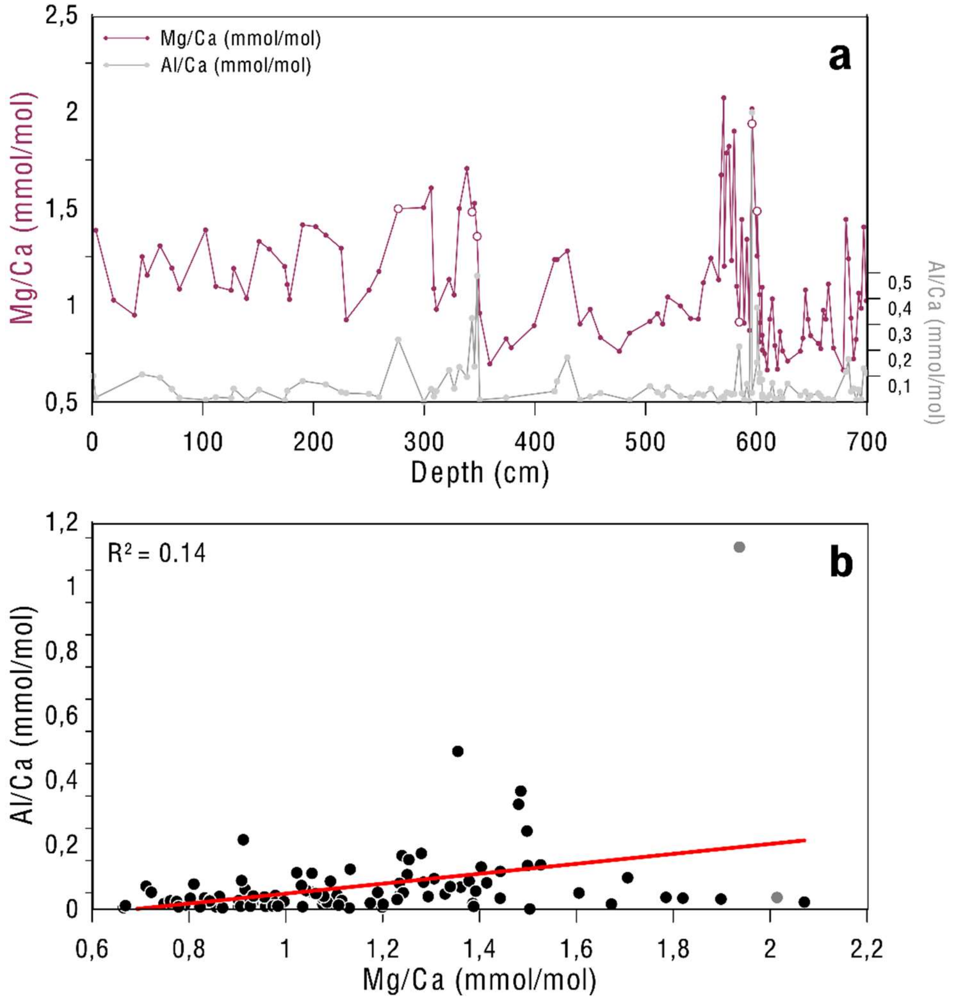

Supplementary Fig. 1 Quality control for Mg/Ca measurements. **(a)** Al/Ca is used to monitor the potential influence of silicate contamination in foraminiferal Mg/Ca ratios. Al/Ca >0.1 mmol/mol suggest contamination may be significant[40]. Most samples are well below Al/Ca >0.1 mmol/mol. Six samples (open circles) have values above Al/Ca >0.2 mmol/mol but do not appear to have anomalously elevated Mg/Ca ratios. **(b)** Cross-plot of Mg/Ca values versus Al/Ca values showing generally weak correlation between Mg/Ca and Al/Ca (r^2 = 0.14). Grey spots indicate replicate measurements of a sample with highly elevated Al/Ca ratio.

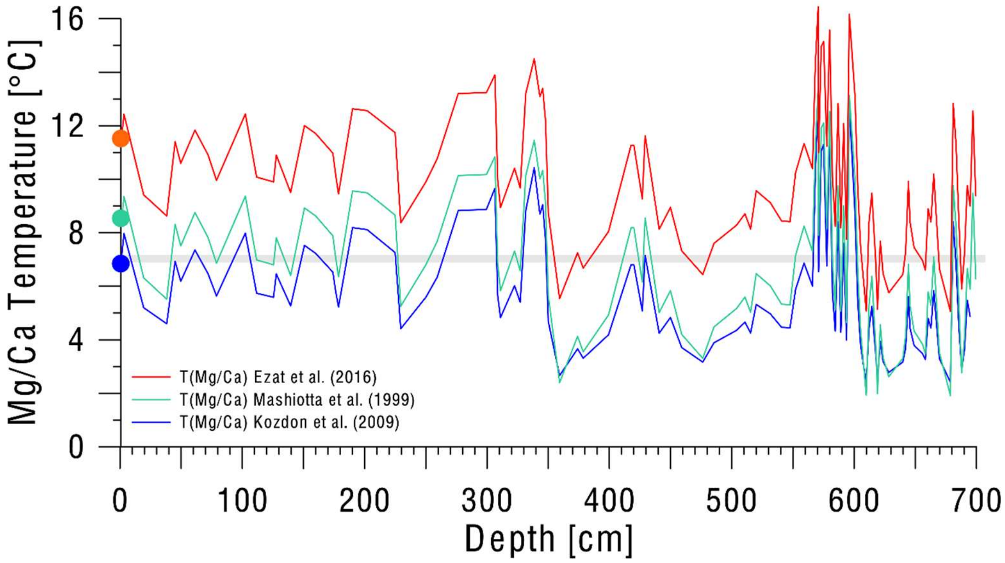

Supplementary Fig. 2 Comparison of different *N.pachyderma* sin. Mg/Ca-temperature calibrations[42-44]. Grey bar indicates modern subSST$_{150m}$[15] near core site GeoB18530-1. Coloured spots indicate core-top Mg/Ca-temperatures for different calibrations.

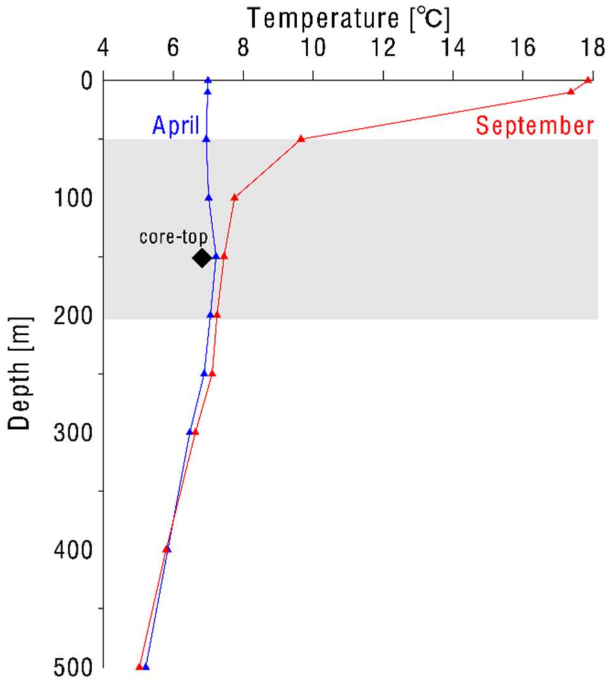

Supplementary Fig 3. Instrumental temperature profiles near site GeoB18530-1. Temperature profile for April (blue) and September (red) close to core site GeoB18530-1[15]. Grey bar indicates modern habitat depth range of *N.pachyderma* sin. in the North Atlantic[43,45]. Black diamond = core-top Mg/Ca temperature of GeoB18530-1 calibrated after ref.43.

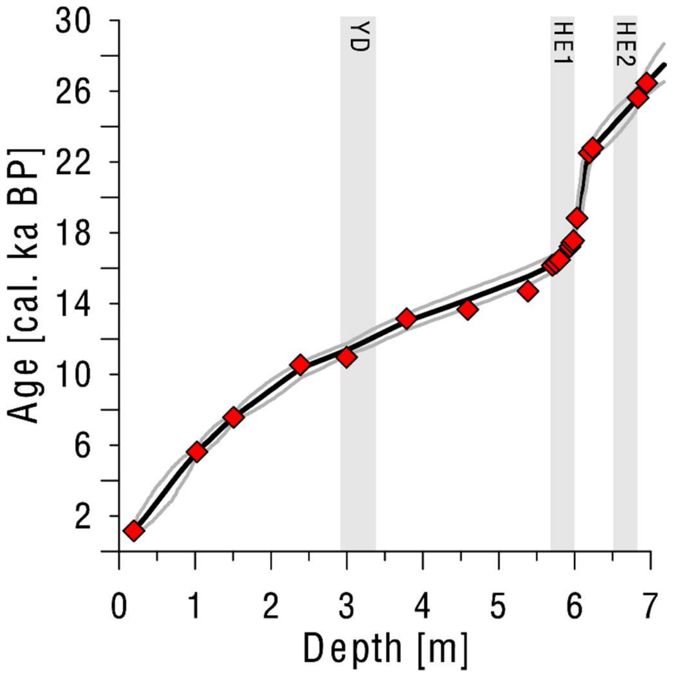

Supplementary Fig. 4 Age model of sediment core GeoB18530-1. Results of age-depth modeling using the R script BACON[51] with mean ages (black line) and 95% confidence intervals (grey lines) for GeoB18530-1. Red diamonds = Age control points from accelerator mass spectrometry radiocarbon datings; Grey bars = Heinrich Layers and YD.

Supplementary Table 1 Radiocarbon ages and derived age model of GeoB18530-1.

List of radiocarbon ages from core GeoB18530-1

Depth [m]	Lab code	Type	^{14}C age raw [ka]	Age error +/- [ka]	Reservoir Age [ka]	lower age [ka] 2σ	upper age [ka] 2σ	Calendar age [ka BP]	Sample type
0,194	Poz-81680	AMS	1,65	0,03	0,445	0,98	1,28	1,13	*N.pachyderma* sin.
1,025	Poz-81681	AMS	5,33	0,035	0,436	5,47	5,88	5,64	*N.pachyderma* sin.
1,508	BE-12553.1.1	AMS	7,20	0,046	0,468	7,43	7,69	7,59	*N.pachyderma* sin.
2,387	Poz-81682	AMS	9,72	0,06	0,359	10,28	11,06	10,57	*N.pachyderma* sin.
2,995	BE-12554.1.1	AMS	10,54	0,049	0,722	10,88	11,61	11,25	*N.pachyderma* sin.
3,786	Poz-81683	AMS	12,33	0,06	0,844	13,07	13,79	13,38	*N.pachyderma* sin.
4,591	Poz-81685	AMS	12,83	0,07	0,781	13,50	14,83	14,01	*N.pachyderma* sin.
5,384	Poz-81686	AMS	13,31	0,07	0,802	14,06	15,36	14,70	*N.pachyderma* sin.
5,706	BE-12555.1.1	AMS	13,96	0,051	0,874	15,15	16,26	15,69	*N.pachyderma* sin.
5,754	BE-12556.1.1	AMS	14,06	0,051	0,875	15,26	16,38	15,82	*N.pachyderma* sin.
5,8	BE-12557.1.1	AMS	14,28	0,06	0,896	15,56	16,74	16,12	mixed planktics (*G. bulloides/N.pachyderm*a sin.)
5,938	BE-12558.1.1	AMS	15,14	0,054	1,002	16,65	17,83	17,22	*N.pachyderma* sin.
5,961	BE-12559.1.1	AMS	15,03	0,054	0,982	16,50	17,72	17,07	*N.pachyderma* sin.
5,984	BE-12560.1.1	AMS	15,40	0,053	1,027	17,05	18,12	17,55	*N.pachyderma* sin.
6,03	BE-12561.1.1	AMS	16,60	0,058	1,02	18,31	19,36	18,87	*N.pachyderma* sin.
6,191	BE-12562.1.1	AMS	20,19	0,084	1,014	22,68	23,75	23,21	*N.pachyderma* sin.
6,237	Poz-81687	AMS	19,97	0,12	1,05	22,38	23,67	22,82	*N.pachyderma* sin.
6,833	BE-12563.1.1	AMS	22,30	0,099	1,204	24,98	25,87	25,44	*N.pachyderma* sin.
6,948	Poz-81689	AMS	24,09	0,18	1,241	26,46	27,66	27,11	*N.pachyderma* sin.
7,5	Poz-81690	AMS	31,20	0,4	1,087	33,81	35,45	34,64	*N.pachyderma* sin.
